# Breaking Boundaries: First Observation of Intertidal Emergence During Mating in Octopuses

**DOI:** 10.1002/ece3.73503

**Published:** 2026-04-23

**Authors:** Hidde Juijn, Miguel Cabanellas‐Reboredo, Ángel F. González, Jorge Hernández‐Urcera

**Affiliations:** ^1^ Centro Oceanográfico de Illes Balears (COB‐IEO), CSIC Palma de Mallorca Spain; ^2^ ECOBIOMAR Research Group Institute of Marine Research (IIM‐CSIC) Vigo Spain

**Keywords:** *Callistoctopus macropus*, in situ observation, intertidal emergence, reproductive behaviour, sexual conflict

## Abstract

Reproductive behaviour in octopuses is diverse, yet field observations remain limited for nocturnal species such as *Callistoctopus macropus*. We report the first documented case of intertidal emergence during copulation in an octopus. During a night dive in Ibiza, a mating pair of *C. macropus* moved gradually from shallow water onto the rocky shore while remaining physically connected. Once emerged, the female showed a putative escape behaviour, anchoring to nearby rocks, while the male persisted and counter‐anchored, resulting in a prolonged struggle with both individuals partially exposed for ~20 min before re‐submerging. This behaviour is unprecedented and suggests that intertidal emergence may function as an extreme female resistance tactic within the context of sexual conflict. These observations expand the known behavioural repertoire of octopuses and highlight the need for further study of social dynamics in natural settings.

## Graphical abstract

We document the first recorded case of intertidal emergence during copulation in Callistoctopus macropus. A mating pair gradually moved from shallow water onto the rocky shore while remaining attached, with the female resisting and the male counter‐anchoring for nearly 20 min before re‐submerging. This unprecedented behaviour suggests that emergence may serve as an extreme form of female resistance within sexual conflict, expanding the known behavioural repertoire of octopuses.

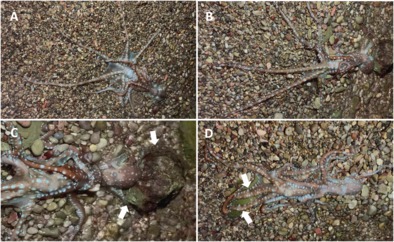

## 1 Introduction

Octopus reproductive behaviour is remarkably diverse, encompassing fleeting encounters, prolonged embraces, and complex mate‐guarding tactics (Hanlon and Messenger 2018). Males transfer spermatophores via the hectocotylus, a modified arm inserted into the female's mantle cavity (Hanlon and Forsythe 2008). While cooperative or non‐aggressive interactions are common, several cephalopod species exhibit behaviours consistent with sexual conflict, understood as the evolutionary divergence of male and female reproductive interests. Documented examples include male attempts to physically restrain females during copulation in *Octopus cyanea* (Huffard and Bartick 2015), mate‐guarding and persistent following of females by males in species such as *Abdopus aculeatus* (Morse and Huffard 2019), and competition among males in squid such as *Loligo bleekeri* , where males adopt coercive or sneaking tactics to increase fertilisation success (Ibáñez and Keyl 2010; Morse and Huffard 2019). These behaviours illustrate how males may impose mating costs on females or limit their ability to choose or avoid partners.

Despite the growing interest in cephalopod reproductive ecology, most knowledge derives from laboratory studies or observations of relatively accessible, shallow‐dwelling species (Rocha et al. 2001; Hanlon and Messenger 2018). Behavioural data collected in situ remain scarce, particularly for nocturnal and cryptic taxa such as *Callistoctopus macropus*. This scarcity hampers our understanding of how environmental complexity, predation risk, and intersexual interactions shape mating strategies under natural conditions. Reports of extreme or unusual reproductive behaviours, whether involving mate guarding, forced copulation, or resistance tactics, are especially valuable because they provide insight into the balance of selective pressures acting on both sexes. Documenting such behaviours in the wild not only broadens our knowledge of cephalopod ethology but also helps refine theories of sexual conflict and reproductive investment in marine invertebrates.

The white‐spotted octopus, *C. macropus* , a relatively understudied species, is a nocturnal benthic species distributed across the Mediterranean and eastern Atlantic, commonly inhabiting rocky reefs, seagrass beds, and mixed substrates from shallow subtidal to ~17 m depth (Norman 2000). To our knowledge, no published records describe *C. macropus* spending extended periods out of water, making the prolonged intertidal emergence reported here unprecedented for this species. Although some intertidal foraging has been noted in related species (Röckner et al. 2024; Yamate et al. 2021), emergence during mating has not been described. Here, we present the first documented case of intertidal emergence during copulation in *C. macropus* , observed in situ, and discuss its potential significance for understanding mating systems and sexual conflict in this species.

## 2 Materials and Methods

The observation was documented on 2 November 2022 at 21:55 local time during a night dive in a small cove near Sant Antoni de Portmany (38.99138889° N, 1.28583333° E) on Ibiza, Balearic Islands, Spain. Depth at initial observation was ~0.8 m, ~5 m from shore. The substrate consisted of rock and cobble‐sized rounded stones in shallower areas. The event was recorded by divers using a Canon EOS 5D Mark II, in a Subal housing and outfitted with two Inon Z240 strobes. Because tidal amplitude in Ibiza is minimal (< 20 cm), the wet rocky substrate resulted from continuous wave wash rather than from a significant tidal change. The event occurred after sunset, under low ambient light conditions and without direct moonlight, so visibility relied primarily on diver lights and low‐intensity strobes. During the observation, divers maintained a respectful distance of approximately 1–2 m to avoid interfering with the natural behaviour of the octopuses. Whenunderwater photographs were taken, the lowest possible strobe intensity was used to minimise disturbance. Species identification was based on diagnostic morphology (reddish‐brown body, elongate arms with white spots, head–mantle proportions; Norman 2000).

## 3 Results

**FIGURE 1:**
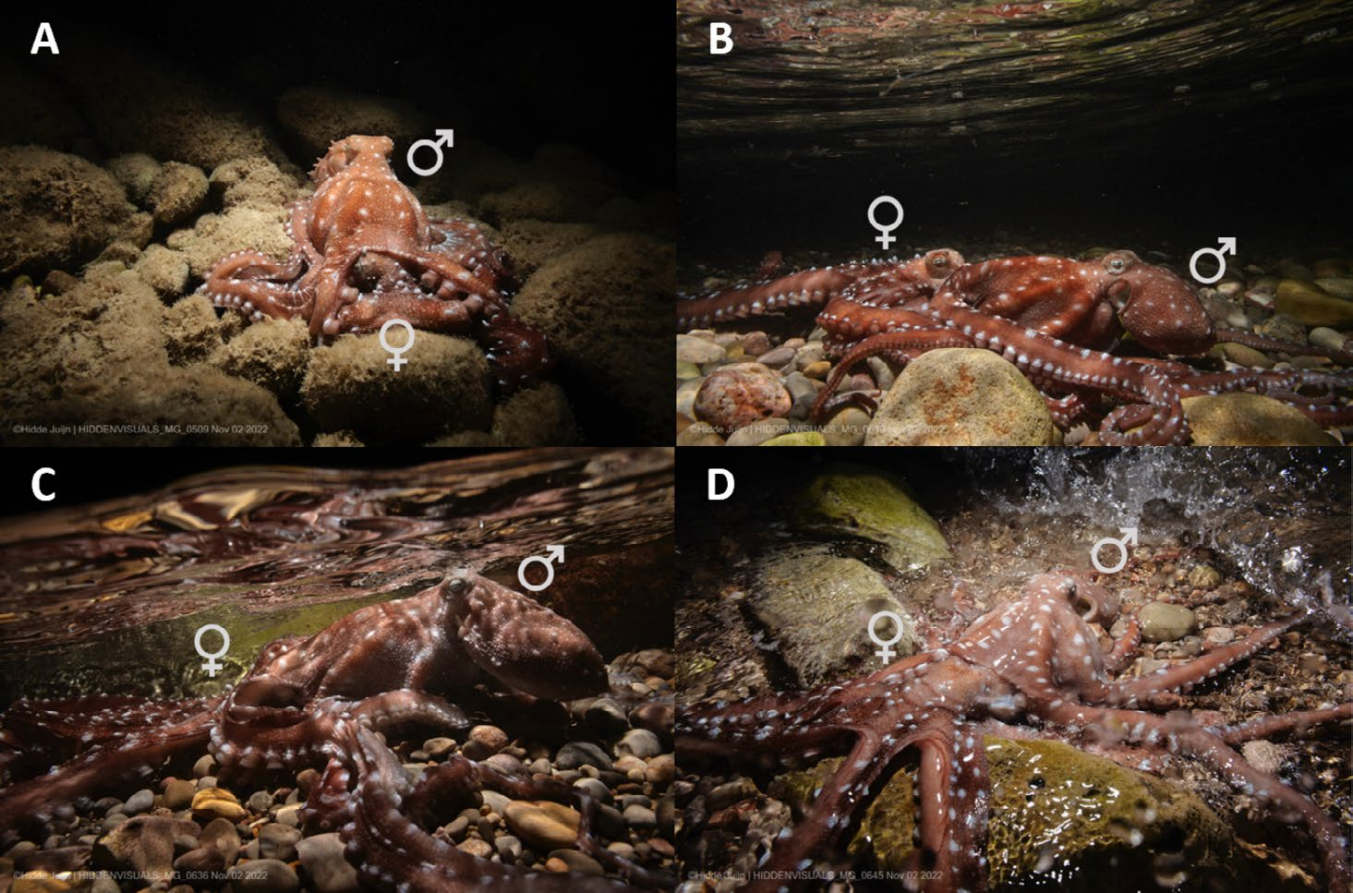
Sequential observations of mating behaviour in *Callistoctopus macropus* (underwater phase). (A) Initial stage of observation showing the male positioned above the female in a typical copulatory posture. (B) The pair advancing toward the shoreline, the male maintaining a firm grasp as the female moved forward. (C) Gradual progression into shallower water, from approximately 0.8 m to less than 0.1 m depth. (D) The female fully emerged onto the interdidal zone while the male remained physically attached.

At the start of the observation, two *C. macropus* individuals were engaged in close physical contact. Although no images are available to confirm it, divers reported that the individual positioned on top had its third right arm inserted into the mantle cavity of the individual beneath, identifying it as the male and the lower individual as the female. The male maintained close body contact with several arms wrapped around the female while adopting the characteristic dorsal‐mounting position used for spermatophore transfer (Figure 1A). This position was maintained for approximately 35–40 min underwater. During this period, the female gradually moved closer to the shoreline despite the male remaining firmly attached (Figure 1B). The pair progressed toward shallower depths, moving from approximately 0.8 m to less than 0.1 m (Figure 1C). Eventually, the female emerged completely from the water onto the intertidal zone, with the male still physically connected (Figure 1D). During this emersion, both individuals supported their own weight.

**FIGURE 2:**
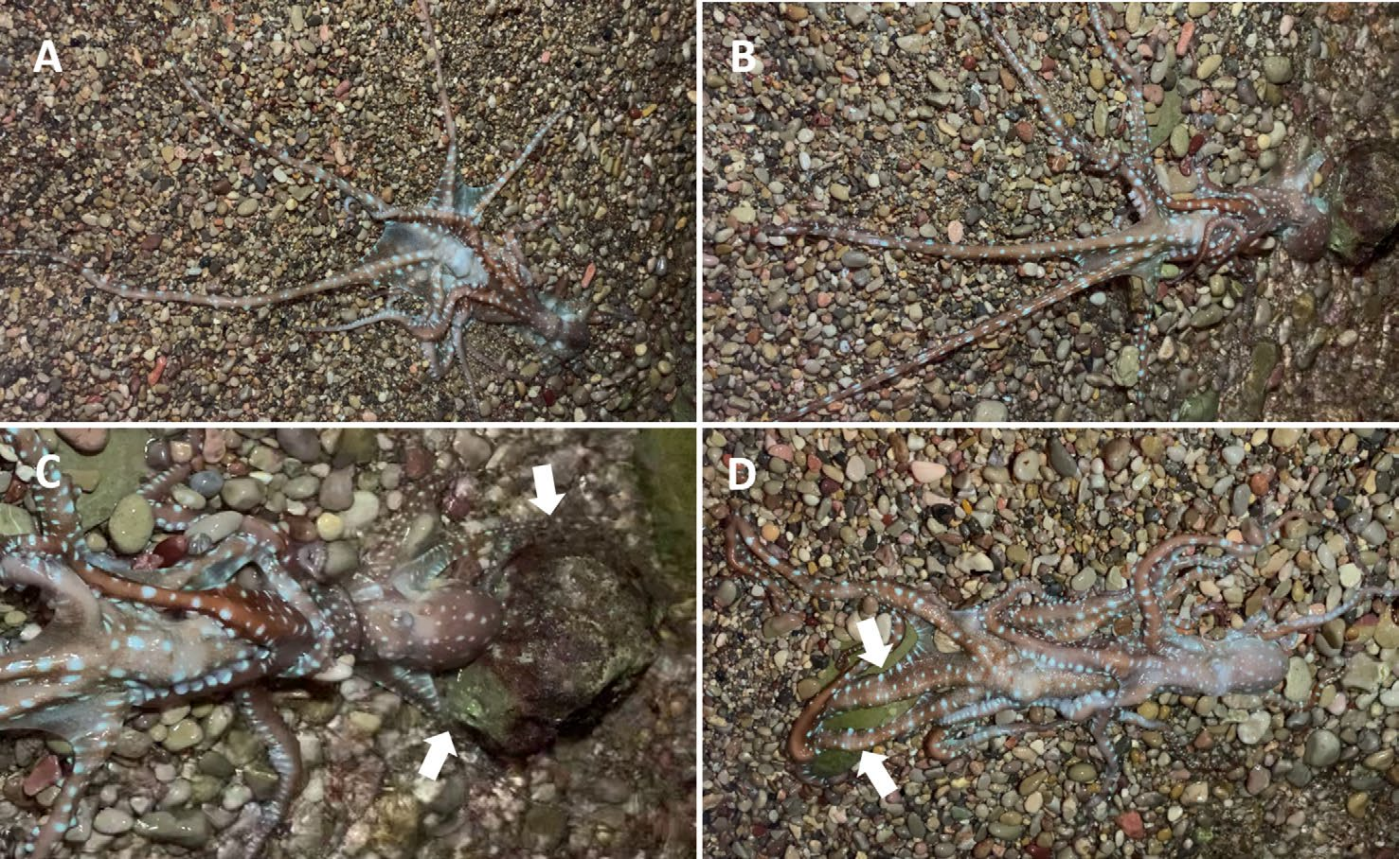
Continuation of the observed mating event in *Callistoctopus macropus* (intertidal phase). (A) The female (left) attempting to move away from the intertidal zone, dragging the attached male in the opposite direction. (B) The male (right) pulling the female toward a rock anchored to the substrate. (C) The male (right) wrapping two arms (R4 and L4) around the rock (white arrows) to anchor and exert greater pushing force. (D) The female (left) dislodging the male from the rock and performing a similar anchoring manoeuvre (white arrows; L1 and L2) to pull the male toward the upper part of the intertidal zone.

Once in the intertidal zone (Video S1), the female exhibited a putative escape response by moving away from the water, dragging the attached male in the opposite direction (Figure 2A). The pair remained physically connected, but it was not possible to determine whether copulation continued during the emergence. In response, the male pulled the female toward a rock anchored to the substrate (Figure 2B) and wrapped two arms (R4 and L4) around the rock to anchor and exert greater pulling force (Figure 2C). At this point, both came into contact with the water, enabling respiration to resume. The female succeeded in separating the male from the rock and performed a similar anchoring manoeuvre using another rock to pull the male toward the upper part of the intertidal zone (Figure 2D). For about 20 min, both remained partially exposed as waves washed over them, the female resisting the male's retreat to deeper water by anchoring to the rocks with arms and suckers. Eventually, the male succeeded in re‐submerging the female, after which both disappeared. No signs of predation or injury were observed throughout the event.

## 4 Discussion

To our knowledge, no prior account exists of intertidal emergence during or after mating in *C. macropus* , with the male maintaining a prolonged embrace around the female throughout the emergence. While some octopus species have been reported moving over exposed substrates for foraging or to reach neighbouring tidepools (Yamate et al. 2021; BBC 2023), no prior account exists of intertidal emergence after mating. This finding reveals a previously unrecognised behavioural possibility in octopuses and expands our understanding of the diversity of reproductive strategies in cephalopods. The combination of prolonged copulation, shoreward movement, and nocturnal intertidal exposure seems to be uncommon in *C. macropus* and is not well documented in other octopus species.

Octopuses rely primarily on gill respiration and have limited capacity for aerial oxygen uptake through skin and mantle epithelia (Wells and Wells 1983). Although aerial exposure can impose risks such as desiccation and thermal stress, the female's mantle cavity remained intermittently hydrated by wave splash and retained water, making it possible that some level of respiration continued throughout the event. The approximate ~20 min during which the animals were partially or fully above the waterline may still have posed a physiological challenge for both individuals. The female was actively resisting and therefore expending additional energy, while the male maintained a continuous grip and repeatedly pulled her toward the water. Nonetheless, both individuals resumed normal activity without visible impairment, suggesting that *C. macropus* may possess short‐term tolerance to intertidal emersion comparable to that reported in other intertidal octopuses (Röckner et al. 2024).

Several potential predators of small to medium‐sized octopuses occur at this site, including nocturnally active moray eels ( *Muraena helena* ), conger eels ( *Conger conger* ), and scorpionfish (*Scorpaena* spp.) in subtidal areas. Potential terrestrial or semi‐terrestrial predators include nocturnally active herons such as *Nycticorax nycticorax* , as well as opportunistic mammals (e.g., feral cats or rats), which could attack exposed individuals in the intertidal zone. Although no predators were observed during the event, the combination of nocturnal timing and partial emersion would have placed the animals within reach of predators from both the subtidal and intertidal environments.

Given that this report is based on a single opportunistic observation, any interpretation of function must remain tentative. Several explanations, including accidental displacement, avoidance of a perceived threat, or cannibalism avoidance, cannot be ruled out. However, interpreting this behaviour as resistance remains speculative and cannot be confirmed from a single observation. Because it is a single event, the functional significance of the anchoring behaviour remains uncertain and multiple interpretations should be considered. A plausible explanation for the observed partial emergence onto the rocky shore is that it represents an extreme escape tactic by the female. Sexual conflict theory predicts that when mating costs to females are high, due to physical injury risk, energy expenditure, predation vulnerability, or interference with future reproductive opportunities, selection may favour strong resistance behaviours (Clutton‐Brock and Parker 1995; Arnqvist and Rowe 2005). Similar forms of sexual conflict have been documented in other octopus species, including male‐induced envenomation in *Hapalochlaena fasciata* (Chung et al. 2025) and constricting behaviours in *Octopus cyanea* and *Wunderpus photogenicus* (Huffard and Bartick 2015), highlighting that coercive or harmful interactions can occur during mating in this group. Choosing to move into an environment with elevated mortality risk suggests that the perceived cost of continued copulation outweighed the dangers of aerial exposure. Similar high‐risk escape behaviours have been documented in other taxa, such as female insects diving into water to evade males (Miller 2003).

The male's pursuit into the intertidal zone is equally striking. By moving partially out of the water and onto the exposed intertidal substrate, the male accepted the same suite of risks as the female, indicating strong selective pressure for mate retention. In cephalopods, male persistence can involve blocking the female's den entrance, physically restraining her arms, or engaging in prolonged mate guarding to ensure sperm precedence (Huffard and Godfrey‐Smith 2010; Hanlon and Messenger 2018). Following a mate onto land adds a novel dimension to male persistence, suggesting that, at least in some contexts, the drive to secure copulation may override even substantial immediate mortality risks. Comparable extremes occur in other cephalopods: for example, male *Octopus tetricus* frequently engages in prolonged grappling and sustained physical contests over access to females, incurring high injury risk (Scheel et al. 2016). In *Loligo pealeii* , males maintain close contact with females during intense mating aggregations where predation risk is elevated (Hanlon et al. 1997). Similarly, male *Sepia apama* remain in exposed shallow breeding grounds for extended periods, where they face increased predation and energetic costs while guarding females (Hall and Hanlon 2002). These parallels illustrate that strong sexual competition can drive males to accept considerable danger.

Both male and female using rocks to anchor themselves during the intertidal struggle underscores the remarkable adhesive strength of octopus suckers. These suckers generate considerable suction forces, enabling strong attachment to irregular and hard substrates even under tension (Bagheri et al. 2020). This adhesive capacity likely facilitates the anchoring manoeuvres observed, where the male wrapped two arms around a rock to enhance leverage and resist the female's putative escape response, while the female subsequently employed a similar strategy with another rock to counter the male's force. Such use of environmental features to augment physical resistance has been documented in other octopus species during agonistic or mating interactions (Kier and Smith 2002; Packard 1988). The ability to exploit substrate heterogeneity for mechanical advantage may be crucial in the intertidal zone, where complex rocky habitats provide varied anchoring points.

There is limited knowledge regarding how this species reproduces in the wild. Recent work on the reproductive biology of the species (Riad 2024) has characterised maturity stages, fecundity patterns and spawning seasonality in *C. macropus* , but behavioural observations in the wild remain extremely scarce. Our observation provides the first detailed account of a male mounting a female in situ, revealing a prolonged embrace of approximately 35–40 min, during which the male maintained multiple arms in contact and inserted the hectocotylus into the female's mantle cavity. This represents the first detailed account of intertidal emergence during copulation specifically for *C. macropus* . Such prolonged copulatory behaviour resembles patterns reported in other octopus species, such as *Octopus bimaculoides* , *O. cyanea* , *O. briareus* and *O. vulgaris* (Hanlon and Messenger 2018). While the escape hypothesis is consistent with observed resistance, alternative explanations merit consideration. For example, displacement shoreward by environmental factors such as currents or predator avoidance could have occurred, but conditions were calm, no predators were observed, and the female's post‐copulatory movement was gradual and deliberate rather than consistent with forced drift. Although diver presence cannot be entirely excluded as a potential influence, several factors suggest that the emergence was not a response to the observers. The pair was already mating when first detected, and their progression toward shallower water was continuous while divers maintained a distance of ~1–2 m and avoided strong direct illumination. If the individuals had been attempting to avoid the observers, they could have moved toward deeper water rather than continuing into the intertidal zone, and neither showed behavioural signs typically associated with disturbance (e.g., rapid colour change, jetting, or sudden displacement).

This observation highlights the value of opportunistic documentation in revealing cryptic marine animal behaviour. Systematic surveys in shallow habitats, especially at night and during transitional tides, could help determine the frequency of such events. Additionally, controlled studies on aerial tolerance and energetic costs of intertidal excursions in *C. macropus* would clarify physiological trade‐offs. Understanding triggers and consequences of such extreme behaviours will improve models of sexual selection and conflict in cephalopods (Hanlon and Messenger 2018; Arnqvist and Rowe 2005).

## Author Contributions

**Hidde Juijn:** conceptualization (equal), data curation (equal), formal analysis (lead), investigation (equal), methodology (lead), visualization (equal), writing – original draft (equal). **Miguel Cabanellas‐Reboredo:** conceptualization (equal), investigation (equal), supervision (supporting), writing – review and editing (equal). **Ángel F. González:** conceptualization (equal), funding acquisition (equal), investigation (equal), project administration (lead), writing – review and editing (equal). **Jorge Hernández‐Urcera:** conceptualization (equal), data curation (equal), formal analysis (supporting), funding acquisition (equal), investigation (equal), supervision (lead), visualization (equal), writing – original draft (equal), writing – review and editing (equal).

## Funding

This research was supported by funding from the Ministry of Science, Innovation and Universities, Spain/ECOSUMA Project (PID2019‐110088RB‐I00).

## Ethics Statement

This is an observational study initiated from observations in the wild without disturbing the animals. The IIM‐CSIC Research Ethics Committee has confirmed that no ethical approval is required.

## Conflicts of Interest

The authors declare no conflicts of interest.

## Supporting information


**Video S1:** Continuation of the observed mating event in *Callistoctopus macropus* during the intertidal phase.

## Data Availability

The original contributions presented in the study are included in the article or Supporting Information (Video S1). Further inquiries can be directed to the corresponding author.
